# Mapping of actionable mutations to histological subtype domains in lung adenocarcinoma: implications for precision medicine

**DOI:** 10.18632/oncotarget.1840

**Published:** 2014-03-22

**Authors:** Gavin M. Wright, Hongdo Do, Jonathan Weiss, Naveed Z. Alam, Vivek Rathi, Marzena Walkiewicz, Thomas John, Prudence A. Russell, Alexander Dobrovic

**Affiliations:** ^1^ University of Melbourne Department of Surgery, St Vincent's Hospital Melbourne, Victoria, Australia.; ^2^ Translational Genomics and Epigenomics Laboratory Ludwig Institute for Cancer Research Olivia Newton-John Cancer and Wellness Centre Heidelberg, Victoria, Australia.; ^3^ Department of Anatomical Pathology St Vincent's Hospital Melbourne, Victoria, Australia.; ^4^ Ludwig Institute for Cancer Research, Olivia Newton-John Cancer and Wellness Centre Heidelberg, Victoria, Australia.; ^5^ Department of Pathology, University of Melbourne, Melbourne, Victoria, Australia.

**Keywords:** BRAF, EGFR, KRAS, high resolution melting, polymerase chain reaction, tumor heterogeneity.

## Abstract

Precision medicine depends on the accurate identification of actionable mutations in a tumor sample. It is unknown how heterogeneous the distribution of such mutations can be in a tumor. Morphological (i.e. histopathological) heterogeneity is well described in lung adenocarcinoma and has been specifically recognized in the most recent official clinico-pathological classification. The most predominant subtype present is now used to classify each lung adenocarcinoma. No molecular profile exists to explain the intratumoral differences in lung adenocarcinoma morphology, despite the consistently observed association between specific predominant subtypes and poorer survival. Given a recent proposal stratifying lung adenocarcinoma into subtypes of differing metastatic potential, we questioned the assumption that major mutations are present uniformly throughout tumors; especially those showing discrete different subtypes.

We selected formalin-fixed paraffin embedded lung adenocarcinoma specimens that showed discrete areas of different subtypes, extracted subtype DNA samples from those areas and screened for mutations in hotspot regions of the *EGFR*, *KRAS* and *BRAF* genes using high resolution melting. Sanger sequencing was used to confirm all identified mutations. Chromogenic *in situ* hybridization (CISH) was used to identify mutant allele specific imbalances in tumors with *EGFR* mutations.

Interestingly, we found that *KRAS* and *BRAF* mutations could be confined to morphological domains of higher grade. On the other hand, *EGFR* mutations were found through all histological subtypes in each tumor consistent with the driver status of this mutation.

Intratumoral heterogeneity has major implications for tumorigenesis, chemoresistance and the role of histopathology in molecular screening for precision medicine. This study not only confirms that intratumoral mutational heterogeneity does occur, but also that it is associated with morphologically distinct regions in some tumors. From a practical perspective, small biopsies may not adequately represent a tumor's full mutational profile, particularly for later arising but prognostically important mutations such as those in the *KRAS* and *BRAF* genes.

## INTRODUCTION

Tumor heterogeneity has different meanings in different clinical disciplines. To the anatomical pathologist, it is related to observable histologic differences in regions of a tumor; to the molecular pathologist, it relates to mutational variation between cells of the same tumor; and to the medical oncologist, it is related to therapeutic response. Few cancers demonstrate tumor heterogeneity as clearly as lung adenocarcinoma. Morphologically, lung adenocarcinomas are typically heterogeneous, with in excess of 90% of resected tumors comprised of multiple histologic subtypes [1-3].

The existence of intratumoral mutational heterogeneity in lung adenocarcinoma has been debated since activating *EGFR* mutations were found to predict response to EGFR tyrosine kinase domain inhibitors. It is clinically evidenced after the selective pressure of effective chemotherapy or molecular targeted therapy when tumors later progress. Whilst Jiang *et al* reported evidence indicating *EGFR* mutational heterogeneity in at least one patient [4], Yatabe *et al* concluded that for *EGFR* mutations, this was likely to be non-existent, or at least exceedingly rare. The conclusions of Yatabe *et al* were based on multiple random samples from five *EGFR* mutant lung adenocarcinomas without reference to areas of variation in intratumoral morphology. Their hypothesis was that previously reported cases of intratumoral mutational heterogeneity were false negative results associated with low tumor purity, insensitive direct sequencing techniques and pseudo-heterogeneity caused by variation of mutant allele copy number across the tumor [5]. A subsequent Korean study has examined *EGFR* mutations in different subtypes of ten adenocarcinomas and found no evidence of heterogeneity in the two to three selectively dissected tissues from each tumor [6].

The concept of branching evolution, where all cells in a given tumor harbor common truncal mutations, but different parts of the tumor (or its metastases) develop diverging branch mutations, is now more or less axiomatic, courtesy of evidence from next generation sequencing [7]. However, a corresponding morphological phenotype has not been identified for these divergent genotypes.

In 2011, the International Association for the Study of Lung Cancer, the American Thoracic Society and the European Respiratory Society presented a thoroughly revised lung adenocarcinoma classification [3]. It recognizes the following common histologic patterns or subtypes - lepidic (cancer cells growing along but not invading through alveolar walls), acinar (glandular structures invading adjacent stroma), papillary (branching structures of fibrovascular stroma covered by invasive cancer cells), solid (sheets or nests of cancer cells containing cytoplasmic mucin) and the newly added micropapillary subtype (papillary tufts of tumor cells without fibrovascular cores either lying apparently free in alveolar spaces or surrounded by thin fibrous septa, often at a tumor's edge). A single subtype in a tumor is rare, and any or all the above subtypes can be present in the same tumor. Up to now, there has been no understanding of the molecular basis, if any, of these subtypes.

A study by Sica *et al* suggested that the predominant histologic patterns present in resected lung adenocarcinomas could be equated with tumor grade (N.B. there has never been an internationally recognized grading system for resected lung adenocarcinoma). Based on metastatic potential they concluded that the lepidic pattern correlates with grade 1 (low metastatic potential); acinar and papillary patterns with grade 2 (moderate metastatic potential); and micropapillary and solid with mucin patterns with grade 3 (high metastatic potential) [8].

With its current model of companion testing of a genomic target and therapy with the corresponding inhibitor, effective precision medicine is dependent on finding any actionable mutation. This may be a negative or a positive predictor. In view of the prognostic influence of subtype on survival [1] and the possibility of corresponding molecular heterogeneity, we investigated whether the distinct histologic subtype domains in lung adenocarcinoma harbor important genomic differences.

## RESULTS

### Mutation testing

Twenty-nine adenocarcinoma specimens were considered suitable for the study, having discrete regions of different subtypes that could be targeted by core punch for extraction of DNA. Two tumors were lepidic predominant, eleven were acinar predominant, three were papillary predominant, five were solid predominant and eight were micropapillary predominant. Twenty-six of the 29 tumors had higher grade micropapillary and/or solid components that could be cored for DNA extraction in addition to one or more lesser grade subtypes.

Tumors were screened for mutations in the hotspots of the *EGFR*, *KRAS*, *BRAF* and *TP53* genes. In 24 of the tumors we identified mutations in one or more of the above genes; one of these being a case of pleomorphic carcinoma (50% giant cell/spindle cell, 30% adenocarcinoma, 20% squamous cell carcinoma) with an *EGFR* mutation and another a pleomorphic carcinoma comprising solid, micropapillary and giant cell components with heterozygous allelic *KRAS* mutations of codon 12. Five additional FFPE tumors had none of the common mutations on screening, but had well-demarcated domains of at least two histologic subtypes (table 1). A total of 63 histologic domains of differing subtypes were sampled.

Table 1Morphologic and molecular characteristics of the 29 resected lung adenocarcinoma samples*Bold type denotes samples with intratumoral mutational differences mapping to histologic subtypes of higher metastatic potential. #Denotes excess copy number of *EFGR* mutation present only in the micropapillary subtype. ϕDenotes pleomorphic component of tumor with giant cell and/or spindle cell morphology.

**Table:** 

Patient Tumor Sample	Subtypes screened	*EGFR*	*KRAS*	*BRAF*
1	Acinar Micropapillary	-	G12D G12D	-
2	Acinar Micropapillary	Exon 19 del Exon 19 del	-	-
3	Acinar Micropapillary	Exon 19 del Exon 19 del	-	-
4	Acinar Micropapillary	Exon 19 del Exon 19 del	-	-
5*	**Micropapillary** **Acinar**	**-**	**-**	**V600E** **WT**
6	Acinar Solid Papillary	-	-	-
7	Acinar Lepidic	-	G12V G12V	-
8	Acinar Solid Papillary	L858R L858R L858R	-	-
9	Pleomorphicϕ Acinar Micropapillary	L858R L858R L858R#	-	-
10	Lepidic Micropapillary	-	-	-
11	Lepidic Micropapillary	Exon 19 del Exon 19 del	-	-
12	Lepidic Micropapillary	-	-	-
13	Lepidic Micropapillary	L858R L858R	-	-
14	Lepidic Papillary	-	G12C G12C	-
15*	**Lepidic** **Acinar**	**-**	**WT** **G12C**	**WT** **K601N**
16	Papillary Micropapillary	Exon 19 del Exon 19 del	-	-
17	Papillary Micropapillary	Exon 20 ins Exon 20 ins	-	-
18	Papillary Micropapillary	-	-	-
19*	**Papillary** **Micropapillary** **Solid**	**-**	**WT** **WT** **G12S**	**-**
20	Papillary Micropapillary	-	-	G469V G469V
21*	**Papillary** **Micropapillary**	**-**	**WT** **G12V**	**-**
22	Papillary Micropapillary	-	G13D G13D	G464V G464V
23	Solid Micropapillary	-	-	-
24	Solid Micropapillary	-	-	-
25	Solid Micropapillary	-	-	-
26	Solid, Micropapillary, Pleomorphicϕ	-	G12R/G12C G12R/G12C G12R/G12C	-
27	Solid Acinar	-	G12C G12C	-
28	Micropapillary Acinar	-	G12C G12C	-
29	Micropapillary Papillary	-	G12C G12C	-

All mutations identified by HRM, were confirmed by Sanger sequencing. Nine tumors were found to have *EGFR* mutations (five with exon 19 deletions, three with L858R point mutations and one with an exon 20 insertion). Two of the tumors with exon 19 deletions were co-mutated with *TP53*. Eleven tumors were found to have *KRAS* mutations (ten at codon 12 and one at codon 13) and two of these had co-mutations in *BRAF* (one exon 11 and one exon 15). A total of four tumors (including those co-mutated with *KRAS*) had *BRAF* mutations (G464V and G469V in exon 11, and V600E and K601N in exon 15). Five tumors were mutated in *TP53* hotspots (all different), including the two co-mutated with *KRAS* and one co-mutated with *BRAF* V600E. Mutations in *EGFR* were mutually exclusive with mutations in *KRAS* or *BRAF*. Five tumors were wild type for the four genes tested.

### Mutation status by histologic subtype domain

Out of the 24 tumors with at least one confirmed mutation, intratumoral mutational heterogeneity mapping to a specific histologic subtype was discovered in four cases. Whilst none of the nine *EGFR* mutated tumors were found to be heterogenous, three of eleven *KRAS* mutated tumors (27%) and two of four *BRAF* (50%) were. In all such cases the mutation was present in a higher grade subtype than the wild type component within the same tumor.

In the eleven tumors with known *KRAS* codon 12 or 13 mutations, three were wild type in the predominant subtype, but *KRAS* mutant in a different morphologic subtype of higher grade. The three tumors all had a point mutation in codon 12 (G12C, G12S and G12V). In one of the predominant papillary adenocarcinomas, the papillary and micropapillary subtypes were *KRAS* wild type, but a *KRAS* mutation was present in the solid with mucin subtype. This solid-with-mucin component was the subtype found to be invading both pleura and lympho-vascular spaces, despite being the minority pattern. The patient had pleural recurrence at 8 months and died 2 years later. A second patient with *KRAS* wild type papillary predominant tumor and *KRAS* mutant micropapillary subtype had no evidence of disease after 1 year of follow-up. The percentages of these two tumors comprised of the *KRAS* mutant subtype were in the range of 35-40%. The third such tumor with mutational heterogeneity was in a patient with a good prognosis lepidic predominant adenocarcinoma. This tumor was *KRAS* wild type in the lepidic subtype but *KRAS* mutant in the minority acinar subtype. There was also a co-mutation in *BRAF* (K601N) in the acinar component. This patient survived 10 years without evidence of relapse.

**Fig 1:**
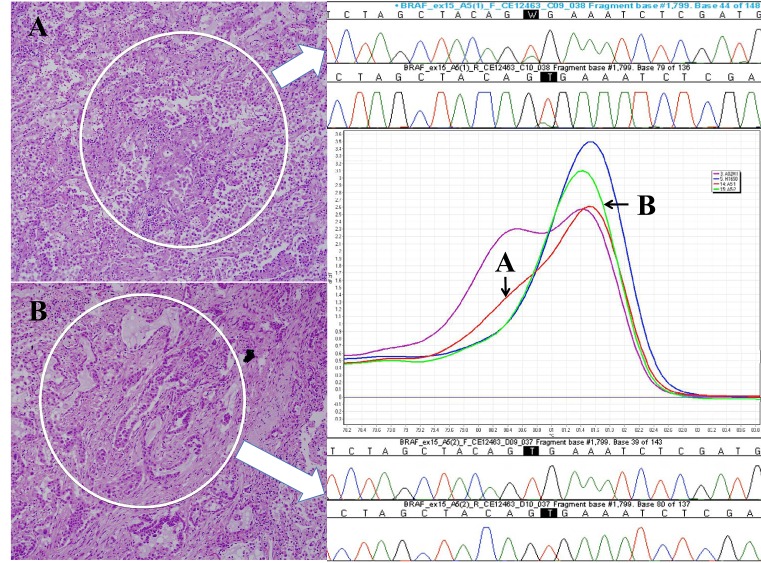
Photomicrographs of the cored areas marked with a dotted circle A, Micropapillary pattern; B, Acinar pattern (H&E, x200 magnification). High resolution melting curves generated for BRAF wild type (green) and mutant (red) corresponding to subtype (arrowed). Sanger sequencing trace for each subtype is shown (arrowed). High resolution melting curves for positive control DNA from A02M1 V600E mutant melanoma cell line (magenta) and negative control DNA from H1650 BRAF wild type lung cancer cell line (blue) are also shown.

Of the four primary tumors with *BRAF* mutations, the tumor from one patient was wild type in the acinar pattern but mutant (V600E) in the higher grade predominant micropapillary pattern (fig. 1). The mutant micropapillary subtype was also the dominant pattern present in mediastinal lymph node metastases of this patient. Three areas from two separate lymph nodes were cored, and all were found to be *BRAF* mutant (micropapillary involvement was so widespread in the node that it was not possible to core any pure acinar or solid component). The patient relapsed with malignant pleural effusion two months later and died of metastatic disease 17 months later.

The primary tumor from a second patient was *BRAF* wild type in the predominant lepidic pattern, but mutant (K601N) only in the minority acinar pattern. This was the same tumor mentioned above with a *KRAS* G12V co-mutation in the minority acinar subtype (patient sample 15 in table 1) with the patient surviving 10 years without relapse.

The remaining two tumors had a *BRAF* mutation in exon 11 (G464V and G469V) present in all histologic subtypes tested.

Importantly, all nine tumors with previously identified *EGFR* mutations in exons 19, 20 or 21 had the relevant mutation identified in all histologic subtype domains tested, i.e. no intratumoral mutational heterogeneity was identified with respect to *EGFR* mutations consistent with an early origin of this mutation.

### Mutant Allele-Specific Imbalance (MASI)

Whilst no differences were detected in *EGFR* mutations between histologic subtypes, a marked imbalance of copy number of the mutant allele was identified in one tumor (patient 9 in table 1). The mutant allele copy number, as assessed by chromogenic *in situ* hybridization, was very high in the micropapillary subtype, but normal in the acinar subtype and pleomorphic regions of the same tumor. No other instances of MASI were identified that mapped to histologic domains.

## DISCUSSION

Intratumoral mutational heterogeneity has been reported in renal cell carcinoma [11], colorectal carcinoma [12] and breast carcinoma [13]. However mutational heterogeneity mapping to a histologic phenotype has only been reported in uncommon biphasic lung cancers. In a study of pleomorphic carcinomas of the lung, one out of six cases had a *KRAS* mutation detected only in the adenocarcinoma component [14]. Pleomorphic carcinomas are uncommon biphasic tumors composed of an epithelial component (e.g. adenocarcinoma, squamous cell carcinoma or large cell carcinoma) associated with a spindle- and/or giant-cell component [15]. In another study of *KRAS* and *EGFR* mutations in adenosquamous lung carcinoma, one out of three tumors had an *EGFR* mutation detected only in the glandular (adeno) component and not in the squamous component [16]. This is not surprising as lung adenocarcinomas and lung squamous cell carcinomas have markedly different genomic and gene expression profiles [17,18]. In accordance with Swanton's trunk and branch evolutionary model [7], the two histotypes could have diverged from their pluripotential stem cell origin by the acquisition of an *EGFR* mutation in one subclone (leading to invasive adenocarcinoma), but a different genomic event in the eventual squamous or pleomorphic component.

**Fig 2:**
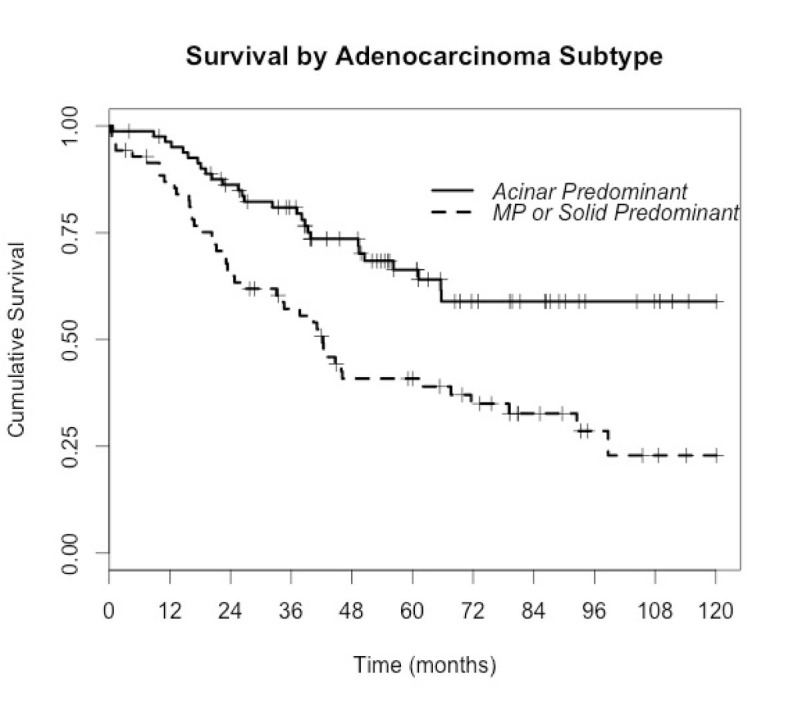
Kaplan-Meier curves (re-plotted from our own data in Russell *et al*, JTO 2011) showing the significantly poorer survival outcomes after surgical resection for micropapillary and solid predominant adenocarcinoma compared to the common acinar predominant (Hazard ratio=2.34, p<0.0005, logrank test). MP=Micropapillary.

We have now provided evidence that intratumoral mutational heterogeneity exists in some lung adenocarcinomas, with additional mutations predictably mapping to a histologic subtype of higher metastatic potential. We propose that the acquisition of a *KRAS* or *BRAF* mutation may drive a more aggressive phenotype that is mirrored by a higher-grade histologic morphology. This could, in some cases, explain the histologic heterogeneity that typifies over 90% of lung adenocarcinoma cases, and the consistent observation that the grade of the predominant subtype is strongly associated with survival outcome (fig. 2) and tumor relapse [1-3,19,20]. *KRAS* mutations, found in about 30% of lung adenocarcinomas [21], are an important negative predictor for *EGFR*, *ALK* and *ROS1* tyrosine kinase inhibitor therapy by virtue of their mutual exclusivity with genomic aberrations of these genes. Although tumor selection in this study was intentionally skewed to those with both mutations and multiple discrete subtype domains, we readily demonstrated intratumoral mutation heterogeneity in three out of eleven *KRAS* mutant tumors. Likewise *BRAF* mutational heterogeneity was found in two of the four tumors suitable for separate subtype sampling. In total, four of 14 tumors (29%) harboring either *KRAS* or *BRAF* mutations were found to have intratumoral mutational heterogeneity with mutations mapping to higher grade histologic subtype domains. Of particular interest is the case of *BRAF* V600E mutation found in micropapillary subtypes in the primary and metastatic nodes but not in the acinar subtype of the primary.

Even if there were very low levels of mutation below the sensitivity of our methodology, the result remains significant, as the tumor purity was high and, by selection and study design, roughly equivalent between the subtype samples from the same tumor. Our mutation screening identified the *EGFR* mutation in all morphologic patterns of all tumors tested, including a single case of pleomorphic carcinoma with MASI demonstrated on *EGFR* chromogenic *in situ* hybridization. This may be an alternative form of progression or resistance in such tumors.

We believe that these findings have far reaching biological and clinical implications not only for lung cancer, but also for tumors in general. Our results call into question the suggestion that *KRAS* and *BRAF* mutations are always early events in the evolution of lung adenocarcinoma, consistent with the conclusion of Sugio *et al* that *KRAS* can be a late event in the pathogenesis of lung cancer [22]. This is difficult to reconcile with the observation that atypical adenomatous hyperplasia (AAH), the presumed dysplastic precursor of adenocarcinoma, has a higher incidence of *KRAS* mutation than adenocarcinoma-*in-situ* and much higher than invasive adenocarcinoma [23]. This suggests that *KRAS* mutated AAH may not progress to adenocarcinoma in the same way proposed for *EGFR* mutated AAH [23-25], and that *KRAS* mutations occur in adenocarcinoma *de novo* via an alternative pathogenesis [26].

The results shown here are reminiscent of the tumor evolution model proposed by Fearon & Vogelstein [27] for colorectal cancer in which *KRAS* mutations were a later change in the tumor. Our results support this for some lung adenocarcinomas, particularly in the patient with lepidic predominant adenocarcinoma. A tumor of pure lepidic component is now considered adenocarcinoma-*in-situ* [3], so the fact that *KRAS* mutations were identified only in the minority invasive acinar component and not in the lepidic component suggests an evolutionary event in this patient.

Histologic subtype domains with aggressive phenotypes such as the micropapillary pattern are not limited to lung adenocarcinoma. They are also seen in breast [28,29], urothelial [30,31], gastric [32,33] and colorectal carcinoma [34,35]. Two studies reported differences in DNA ploidy in the micropapillary component of urothelial carcinoma [30,31]. That fact that the presence of even small foci of this subtype has been reported to have an association with metastatic potential and poor outcome could suggest that the association of more malignant morphological subtypes with additional genetic events found in this study may be generalizable to other solid tumors.

Another consideration raised by these findings is the sensitivity of clinical mutation screening. In the case of *KRAS* or *BRAF* mutant tumors, it may not just be admixture of stromal cells that causes a false negative result on mutation testing. Rather, it may be the presence of tumor cells of a lesser grade morphologic subtype all being wild type or diluting the mutant allele percentage below test sensitivity.

These findings underline the importance of the role of comprehensive histologic subtyping as precision medicine for lung cancer advances beyond its current focus on *EGFR* and *ALK* inhibition. There is increasing dependence on fine needle aspiration cytology and fine core biopsies to minimise patient discomfort. As we found that some of the mutant *KRAS* or *BRAF* domains comprise the minority of the primary tumor, this finding also casts some doubt as to whether small biopsies and cytological specimens from the primary tumor are sufficient to reliably detect mutations other than the ubiquitous *EGFR*.

Another possible implication for the future of precision medicine is that even if newer targeted therapy for *BRAF* or *KRAS* mutations [36] was successfully introduced for lung cancer, it may treat only the more aggressive subtype in some tumors. This could allow the formerly less aggressive phenotype to emerge and progress, or give the appearance of partial response despite excellent biological control of the life-threatening component. As a result of our findings, we would recommend formal incisional or excisional biopsies of primary tumors or sampling of metastases to ensure that all representative subtypes are included in mutation screening.

Our results give further support to evidence that activating *EGFR* mutations are an early genomic aberration in lung adenocarcinoma [5,6,37]. Isolated reports of intratumoral *EGFR* mutational heterogeneity often used less sensitive methods of detection or have been dismissed as MASI, i.e. heterogeneous distribution of mutant *EGFR* amplification within the tumor. One of the *EGFR* mutant tumors demonstrated marked MASI that mapped to the micropapillary subtype. The differential expansion of this clone may represent an escape mechanism from therapeutic tyrosine kinase inhibition. Sequencing of *EGFR* often shows an imbalance in favor of the mutant allele. Based on our results, clinicians can be confident, however, that even small biopsies submitted to HRM screening for *EGFR* mutation will result in appropriate initial therapy.

Lung adenocarcinoma is perhaps the archetype of the heterogeneous tumor. However our findings in lung cancer may have implications applicable to other solid tumors as molecular heterogeneity may not always be reflected by overt histological heterogeneity. As genomic biomarkers become increasingly linked to targeted therapies, the understanding of tumor heterogeneity will become ever more important.

## MATERIALS AND METHODS

### Tumor Tissue Block Selection and Preparation

Four-micron sections were cut from formalin-fixed paraffin embedded tumor blocks of resected adenocarcinomas and stained with haematoxylin and eosin (H&E). Comprehensive histologic subtyping [3] was performed whereby the amount of each adenocarcinoma subtype present in a tumor was estimated in 5% increments followed by classification according to the predominant histologic subtype. Only tumors with discrete areas of at least 2mm diameter of separate subtypes were short-listed for the study so that it was possible to core each independent subtype with a 2mm biopsy punch. The sample set was enriched for mutations by (i) selecting cases that had previously been called as having mutations of *EGFR*, *KRAS*, *BRAF* and/or *TP53* in the clinical or research setting and (ii) selecting tumors with a significant micropapillary subtype component.

The H&E stained sections were used to identify tumors demonstrating different, well-demarcated histologic patterns, with at least 50% tumor cell purity for each pattern. After matching and superimposing each H&E stained section on the glass slide to the surface of the corresponding FFPE tumor block, the specific subtypes of interest were separately sampled with 2mm diameter dermatology core punches. No attempt was made to further microdissect tumor cells, as the intention was to test the full histologic architecture of each particular subtype.

In all tumors, the predominant subtype and at least one other pattern were targeted. We therefore isolated multiple high purity samples of tumors with individual lepidic, papillary, acinar, solid with mucin or micropapillary morphology. Where present in a sufficient quantity, any giant cell or spindle cell patterns of pleomorphic carcinoma were also separately punched. Confirmation of consistent tumor morphology in the Z-plane was achieved by taking a section from the surface of the punched tumor block, staining it with H&E, followed by further cutting at least 1mm deeper into the block and taking another section for H&E staining.

### Deparaffinization and DNA extraction

Using a blade, the punched tumor specimen was diced finely and placed in a screw-cap Eppendorf tube after removing excess paraffin from the underside of the core. Deparaffinization was achieved by treatment with 800 μL xylene, incubation for 5min, vortexing, incubation for 2 min then centrifuging for 1 min at 8000 rpm. This was repeated. Xylene was removed and samples were washed with 800 μL of 100% ethanol. This was centrifuged at 8000 rpm for 1 min and decanted. 800 μL of 70% ethanol was added. This was centrifuged at 8000 rpm for 1 min, with supernatant removed carefully by pipette. Tumor tissue was air-dried in the open on a heat block at 55ºC for 15 minutes.

DNA extraction was achieved using the Qiagen DNeasy Tissue and Blood kit following the manufacturer's instructions with the following modifications: Samples were heated to 98ºC for 15 minutes then incubated at 56ºC for 3 days after addition of 36 μL of proteinase K (Worthington, NJ) at 20 mg/ml concentration.

The eluted DNA sample was stored at 4ºC and concentration was measured using a NanoDrop ND1000 spectrophotometer (Thermo Scientific). Working solutions of 5ng/μl were prepared for use in PCR and sequencing reactions.

Extracted DNA was amplified and scanned for mutations by high resolution melting (HRM) using the assay conditions previously described for *KRAS* and *EGFR* [9]. The region surrounding codon 600 in *BRAF* exon 15 was also screened by HRM using primers tagged with m13 sequences [10] and custom primers were constructed for *BRAF* exon 11 based on known hotspots using the following m13 tagged primers, generating a 143 bp amplicon; forward - 5'-tgtaaaacgacggccagtACTTGGTAGACGGGA CTCGAG-3' and reverse - 5'-caggaaacagctatgaccTGTCACAATGTCACCA CATTACATAC-3'. BRAF exon 11 and exon 15 PCR products were directly used as templates for sequencing reaction. Mutations detected by HRM screening in *EGFR* exons 18-21, *KRAS* exon 2 and *BRAF* exons 11 and 15 were all confirmed by Sanger sequencing.

The St Vincent's Hospital Human Research Ethics Committee approved this study (HREC-A 030/12).
